# A Review on the Neuroprotective Effect of *Moringa oleifera*

**DOI:** 10.1155/2024/7694516

**Published:** 2024-10-24

**Authors:** Beniam Worku, Nafyad Tolossa

**Affiliations:** School of Medicine, College of Health Science, Arsi University, Asella, Ethiopia

## Abstract

*Moringa oleifera*, which is known as a drumstick tree in different areas of the world, is well-known for many health benefits, which are attributed to the abundance of flavonoids, phenolic chemicals, and thiocyanates it contains. This review focuses on *M. oleifera*'s potential for neuroprotection, emphasizing its anti-inflammatory, antioxidant, and neurotransmitter-modulating qualities. Different parts of *M. oleifera* include leaves, roots, bark, and gum. Flowers, seeds, and seed oil are used for many health purposes, most notably in the treatment of neurological diseases. Neurodegeneration, which is characterized by the progressive death of nerve cells, is a major concern with an aging population, leading to disorders such as dementia and movement disorders. *M. oleifera* bioactive compounds improve the antioxidant defense activities of the brain, reduce inflammation, and improve neurotransmitter levels, showing potential therapeutic applications for neurodegenerative disorders. This review emphasizes the importance of further research, especially clinical trials, to fully understand and utilize *M. oleifera*'s neuroprotective capabilities.

## 1. Introduction

*Moringa oleifera*, referred to as drumstick tree, duzigarzan, horseradish tree, benzoilbaumin German, and kelor, contains a wide range of flavonoids and phenolic compounds such as quercetin, kaempferol, rhamnetin, rutin, naringenin, catechin and epicatechin, and isothiocyanates (micronutrients), to which activity has been attributed to the various beneficial health effects of *Moringa* [1]. The tree's leaves, pods, roots, bark, gum, flowers, seed, and seed oil provide a variety of uses that are particularly efficient for health purposes [2]. The consumption of *Moringa* leaf powder can significantly increase the total antioxidant capacity in the body and can lead to inflammation related to chronic diseases as well as decreased damage to DNA, proteins, and lipids [3]. Moreover, the intake of *Moringa* extracts could lead to neuroprotection that can be useful in the treatment of neurodegenerative disease [4]. Furthermore, *M. oleifera* can also improve neurotransmitters due to its significant potentiation of epinephrine and serotonin levels [5].

Neurodegenerative diseases have been an important cause of morbidity and mortality in recent years, and the importance of more effective treatments for these diseases has been defended. Neurodegeneration, the process by which the nervous system degenerates over time, is becoming increasingly common with the elderly population growing [6]. Neurodegenerative disorders can cause many problems, such as dementia, movement disorders, and conditions of thinking, behavior, and mood [7]. Neurodegeneration results from the death of nerve cells throughout the body, whether it is the central nervous system (CNS) or the peripheral nervous system (PNS) [8]. A growing body of evidence currently suggests that oxidative stress, mediated by free radicals, is a major cause of neurodegenerative disorders. Free radicals are atoms or a group of atoms with an odd (unpaired) number of electrons and can be formed when oxygen interacts with certain molecules. Once formed, these highly reactive radicals can start a chain reaction and cause extensive damage to cell constituents. Throughout the nervous system, there are many long-living postmitotic neurons. These neurons have increased time to be exposed to toxins, which makes them more susceptible to oxidative damage [9].

It has been demonstrated that *M. oleifera* may have protective effects on the brain. It has been used to treat and prevent many disorders, including neurological disorders. Among other things, *M. oleifera*'s antioxidant, anti-inflammatory, and neurotransmitter-influencing qualities make it a promising option for research into the treatment of neurological illnesses. This study aimed to review the main studies focused on neuroprotection provided by *M. oleifera* and to encourage future studies in humans, which are very scarce. We found that there is scientific evidence showing the effectiveness of *M. oleifera* in providing neuroprotection, of which the most highlighted benefits are its antioxidant, anti-inflammatory properties, and capacity to balance neurotransmitters.

## 2. Materials and Methods

A comprehensive literature search was conducted to gather relevant studies on the neuroprotective properties of *M. oleifera*. The databases searched included PubMed, Google Scholar, ScienceDirect, and Wiley Online Library. The search terms used were “*Moringa oleifera*,” “neuroprotection,” “antioxidant properties,” “anti-inflammatory properties,” and “neurotransmitter modulation.” The studies included in this review were selected based on the following criteria: published in peer-reviewed journals; written in English; focused on the neuroprotective effects of *M. oleifera*, including its antioxidant, anti-inflammatory, and neurotransmitter-modulating properties; and studies involving in vitro, in vivo, and clinical trials were considered. The exclusion criteria were as follows: studies not focused on neuroprotection, studies not providing detailed methodologies or results, non-peer-reviewed articles, and meta-analyses. Data were extracted from the selected studies regarding the specific neuroprotective mechanisms of *M. oleifera*. This included information on the types of bioactive compounds involved their effects on oxidative stress, inflammation, and neurotransmitter levels, as well as experimental models used (e.g., cell cultures and animal models). The extracted data were synthesized to provide a comprehensive overview of the neuroprotective properties of *M. oleifera*. This synthesis included summarizing the findings on the biochemical pathways influenced by *M. oleifera*, the specific neuroprotective effects observed, and the potential therapeutic applications for neurodegenerative diseases. The quality of the included studies was assessed based on their methodology, sample size, and the robustness of their findings. Studies with rigorous experimental designs and statistically significant results were given more weight in the synthesis of evidence.

## 3. Overview of *M. oleifera*

*M. oleifera* (*M. oleifera*, Lam syn. M. pterygosperma, Gaertn [family Moringaceae]) is a tree originally from the western and sub-Himalayan tracts of India, Pakistan, Asia, Africa, and Arabia. *M. oleifera* is currently found in the Philippines, Cambodia, Central America, North and South America, and the Caribbean Islands [10]. It grows in tropical and subtropical regions across the world. *M. oleifera* is a medium-sized tree [11]. It is known for being resistant to drought, growing in areas with both wet and dry climates, and adapting to different kinds of soil [12].

The therapeutic benefits of *M. oleifera* are due to a variety of phytochemical substances, including phenolic acids, alkaloids, flavonoids, saponins, tannins, and terpenoid [12]. Constituents of *M. oleifera* flavonoids induce the expression of synaptic proteins, promoting neurotransmitter release and upregulating synapse formation by increasing synaptotagmin and postsynaptic density protein 95 (PSD-95) [13]. Alkaloids inhibit pyroptosis-induced neuronal death, reducing inflammatory factors [14]. Phenolic acids alter critical enzymes of purinergic signaling and therefore suggest their possible neuromodulator effect [15]. Its beneficial effects in treating hepatic [16–18], pancreatic [19], pulmonary [20, 21], urinary [22–24], and gastrointestinal diseases [25–27] have been documented.

The therapeutic benefits of *M. oleifera* are due to a variety of phytochemical substances, including phenolic acids, alkaloids, flavonoids, saponins, tannins, and terpenoid [12]. Constituents of *M*. oleifera flavonoids induce the expression of synaptic proteins, promoting neurotransmitter release and upregulating synapse formation by increasing synaptotagmin and PSD-95 [13]. Alkaloids inhibit pyroptosis-induced neuronal death, reducing inflammatory factors [14]. Phenolic acids alter critical enzymes of the purinergic signaling and therefore suggest their possible neuromodulator effect [15]. Its beneficial effects in treating hepatic [16–18], pancreatic [19], pulmonary [20, 21], urinary [22–24], and gastrointestinal diseases [25–27] have been documented.

## 4. Importance of Neuroprotection

The importance of neuroprotection is emphasized by the substantial morbidity imposed by neurological injuries and diseases [28]. Specifically, it is important to consider neurodegenerative diseases such as Alzheimer's, Parkinson's disease, amyotrophic lateral sclerosis, and multiple sclerosis which are burdens on patients, their families, and caregivers and also on the healthcare system [29] and not to forget the incidence and prevalence of traumatic brain injuries and spinal cord injuries, which often lead to loss of motor function and sensory impairments [30]. These types of injuries are often common in soldiers, athletes, and, in a domestic setting, the elderly (“slips and falls”) and are a huge burden on the healthcare system [31]. All in all, it is important to develop means to prevent, cure, and alleviate the symptoms of these diseases.

Over the past 20 years, research on the idea of neuroprotection has expanded and is not a novel idea. In particular, pharmaceutical research focuses on the application of substances and medications designed to guard against harm and injury to the CNS and PNS, to preserve brain function, to rescue neurons, and to stop the advancement of injuries that may cause neurodegeneration. In addition to encouraging functional recovery, potential neuroprotective agents should be able to pass through the blood–brain barrier.

## 5. Mechanisms of Neuroprotection

Neuroprotective agents are the ones that can impede the development of injury, such as neurofunction impairment, by preventing changes to the structure and function of the nervous system. For many CNS disorders, such as neurodegenerative diseases, stroke, and trauma, neuroprotection is a well-researched therapeutic approach [32]. The number of progressive, irreversible CNS disorders that cannot be cured and the amount of disability brought on by CNS injury are still rising, even with the development of numerous neuroprotective medications [29, 30]. To solve these issues, alternative neuroprotective agents are therefore desperately needed.

Oxidative stress is known to play a key role in the pathology of many serious diseases, such as neurodegenerative and cardiovascular diseases, diabetes, cancer, inflammation, and aging [33]. An unbalanced production of free radicals and competing antioxidant defenses is known as oxidative stress. Oxidative stress could result in the formation of toxic reactive oxygen species (ROS) such as hydrogen peroxide, organic hydroperoxides, and nitric oxide (NO), superoxide, and hydroxyl radicals [34]. ROS levels below the homeostatic set point can disrupt the physiological role of oxidants in cell proliferation and host defense [35]. Similarly, elevated levels of ROS can also be harmful and lead to cell death or accelerate aging and age-related diseases [36]. Probably because of its high oxygen requirements, the brain appears to be more vulnerable to oxidative distress than other organs [37]. Oxidative stress triggers neurodegeneration and causes mitochondrial dysfunction, leading to apoptotic neuronal cell death [38]. By maintaining redox balance, a number of endogenous and exogenous antioxidants are used to counteract and protect the organism against free radicals. Antioxidant enzymes (like superoxide dismutase [SOD]) and low molecular weight antioxidants (such as ascorbic acid, uric acid [UA], and vitamin E) are two classes of endogenous mechanisms that neutralize ROS [39]. By dismuting superoxide to hydrogen peroxide, which is then converted to water (peroxidases like glutathione peroxidase and peroxiredoxin) or dismutated to water and oxygen, enzymatic antioxidants control the quantity of superoxide [40]. Because it cannot enter the cells, the exogenous SOD, which is found in the extracellular cell space, has no effect on ischemia. Thus, polyethylene glycol–conjugated SOD was made and applied to experimental animals [41] as well as people with head traumas [42].

## 6. Antioxidant Properties

The most important phenolic compounds in the medicinal plant that are associated with antioxidant activities are flavonoids, phenols, tannins, and alkaloids [43]. According to studies, *M. oleifera* exhibits noteworthy antioxidant qualities. Research has demonstrated that the antioxidant properties of *M. oleifera* are facilitated by the presence of bioactive substances such as polyphenol glycosides, rutin, and other antioxidants [44, 45]. The plant's extracts have shown free radical scavenging properties, reducing power, and antibacterial activities, with the ethanol extract in particular showing strong antioxidant potential [46]. Furthermore, because of the presence of phenolic and flavonoid compounds, leaf extracts from *M. oleifera* have been found to exhibit strong antioxidant activity and antibacterial qualities, making them a viable option for therapeutic applications against bacterial infections and oxidative stress [47, 48].

According to one study, the extracts obtained with water, aqueous methanol, and aqueous ethanol showed free radical scavenging capacities and antioxidant activities. All the extracts effectively scavenged peroxyl and superoxyl radicals with similar activities toward the stable DPPH radical. Among the samples, methanol and ethanol extracts exhibited the highest antioxidant activities in the β-carotene–linoleic acid system. The concentration of the extracts correlated positively with their reducing power and antioxidant activity. The results suggest that *Moringa* leaves are a promising natural source of antioxidants, with methanol and ethanol being the most effective solvents for extraction [49]. Another study found the antioxidant effect of *M. oleifera* leaves using various in vitro methods. They analyzed the effects of *M. oleifera* supplementation on antioxidant enzymes (SOD and catalase [CAT]), lipid peroxidation (LPO), and reduced glutathione (GSH) in goats. The result showed that the acetone extract had higher concentrations of flavonoids, flavonols, phenols, and proanthocyanidins compared to the aqueous extract. Both extracts exhibited strong antioxidant activity, with the acetone extract showing a higher inhibitory effect against various radicals. Supplementation with *M*. oleifera increased the activity of GSH, SOD, and CAT and simultaneously reduced LPO. The results suggest that *M*. oleifera has the potential to be a rich source of antioxidants [50]. Another study showed that the *M*. oleifera extract, along with its major components—cryptochlorogenic acid, isoquercetin, and astragalin—significantly reduced the production of ROS induced by H_2_O_2_ in human embryonic kidney 293 (HEK-293) cells. Notably, treatment with isoquercetin led to a significant increase in the mRNA expression levels of antioxidant enzymes, including SOD, CAT, and heme oxygenase 1[51]. According to these results, *M. oleifera* may be used as a natural antioxidant source to prevent oxidative damage.

## 7. Anti-Inflammatory Effects

Another important causative factor in the development of neurological disease is neuroinflammation [52]. Neuroinflammation is triggered by microglia, the resident immune cells of the CNS, which make up 5%–10% of brain cells [53]. As activated microglia can produce a broad spectrum of neurotoxic molecules, including inflammatory cytokines and reactive oxygen intermediates, it has been suggested that anti-inflammatory therapies may provide new targets for the treatment of these diseases [54].

Studies have highlighted the antineuroinflammatory effects of *M. oleifera*, attributing its benefits to compounds like moringin, astragalin, and isoquercitrin [54]. In a study examining the effects of a hydroethanolic *M. oleifera* on RAW 264.7 macrophages generated by lipopolysaccharide (LPS), it was discovered that *M. oleifera* suppressed the levels of NO, prostaglandin E(2) (PGE[2]), interleukin 6 (IL-6), IL-1*β*, tumor necrosis factor alpha (TNF-*α*), nuclear factor kappa ß (NF-κß), cyclooxygenase-2 (COX-2), and inducible NO synthase (iNOS), but it also increased the levels of anti-inflammatory molecules, including IL-10 and Iκß-*α* [55]. The plant's effectiveness in lowering inflammation has been demonstrated by comparisons of its anti-inflammatory properties to those of common drugs like aspirin and indomethacin [56]. Comparable to aspirin, an isothiocyanate-enriched *M. oleifera* seed extract (MSE) has demonstrated a decrease in carrageenan-induced rat paw edema in vivo. Its main isothiocyanate (macrophage inhibitory cytokine-1 [MIC-1]) at 5 μM can dramatically lower inflammatory cytokines in vitro. Furthermore, MIC-1 at a level of 10 μM can also be more effective than curcumin at upregulating the target genes of nicotinamide adenine dinucleotide phosphate (NAD[P]H), quinone oxidoreductase 1 (NQO1), glutathione S-transferase pi 1 (GSTP1), and heme oxygenase 1 (HO-1) for nuclear factor (erythroid-derived 2)-like 2 (Nrf2) [57].

## 8. Modulation of Neurotransmitters

As the research contexts offered indicate, neurotransmitter dysregulation is a critical factor in the pathophysiology of a number of neurological illnesses. A variety of neurological disorders are associated with disruptions in the metabolism of neurotransmitters, namely, those involving amino acids, monoamines such as dopamine and serotonin, and gamma-aminobutyric acid (GABA) [58–60].

Studies have shown that *M. oleifera* has a significant effect on brain neurotransmitters. *M. oleifera* has been shown to have a significant effect on neurotransmitter levels. According to one study, chronic treatment with *M. oleifera* root extract inhibited penicillin-induced seizures and reduced locomotor activity. This same study showed that chronic treatment with *M. oleifera* increased serotonin (5-hydroxytryptamine [5-HT]) levels in the cerebral cortex, midbrain, caudate nucleus, and cerebellum. It also decreased dopamine (DA) levels in the cerebral cortex, midbrain, caudate nucleus, and cerebellum. However, there was no significant change in norepinephrine (NE) levels in the midbrain, cerebellum, and caudate nucleus, although NE levels were decreased in the cerebral cortex [5]. In the study, various doses of *M. oleifera* root extract were tested, specifically 100, 200, 300, 350, 400, and 450 mg/kg. The results indicated that chronic treatment with *Moringa* significantly inhibited penicillin-induced seizures and altered neurotransmitter levels, particularly at higher doses. Notably, the doses of 300 mg/kg and above were effective in increasing 5-HT levels and decreasing DA levels in the brain regions studied. Conversely, the lower doses, such as 100 mg/kg and possibly 200 mg/kg, did not show significant effects on the seizure activity or neurotransmitter levels, suggesting that a threshold dose is necessary to elicit the central inhibitory effects of *M. oleifera*. Another study showed an increased level of acetylcholine in the cortical hippocampal region by inhibiting the activity of acetylcholine esterase after spatial memory deficits induced by scopolamine in mice [61]. In the study, a *M. oleifera*–supplemented diet (SD) was used as the extract for treatment, with three different concentrations administered: 1%, 5%, and 10% of the diet. The treatment lasted for 7 and 14 days, and the mice were pretreated with scopolamine at a dose of 1 mg/kg prior to the daily oral administration of the *M. oleifera* SD.

Another study also investigated that aqueous extract of *M. oleifera* treatment at doses of 300 and 500 mg/kg significantly increased dopamine and serotonin levels in rats compared to those treated with sodium fluoride alone, with the higher dose of *M*. oleifera (500 mg/kg) showing the best results [62]. Taken together, these results point to the possibility that *M. oleifera* regulates neurotransmitter activity and levels, which may help explain some of its neuroprotective properties in different neurodegenerative conditions.

## 9. Conclusions

*M. oleifera* has shown neuroprotective properties through a lot of mechanisms mainly its antioxidant, anti-inflammatory, and neurotransmitter modulation properties (Table 1). These properties are due to its bioactive substances, such as flavonoids, phenolic acids, and isothiocynates. According to many studies, *M. oleifera* can lower LPO, inhibit proinflamatory cytokines, and enhance the activities of antioxidant enzymes. Its ability to alter neurotransmitter levels highlights its neuroprotective effects even more. Due to these properties, *M*. oleifera has a promising therapeutic option for neurological disorders.

## 10. Future Implications

Future research should focus on several key areas to further validate and expand the understanding of *M. oleifera* neuroprotective effects: Extensive clinical trials are needed to evaluate its efficacy and safety in human subjects, translating promising in vitro and animal study results into potential therapeutic applications for humans. More detailed mechanistic studies are required to understand the precise molecular pathways involved, potentially identifying specific bioactive compounds responsible for the observed effects; research should determine the optimal dosage and administration methods to maximize its neuroprotective benefits, including exploring different forms of the plant and their bioavailability; investigating the long-term effects of *M. oleifera* consumption on neuroprotection and overall health will provide insights into its potential as a sustainable therapeutic option; conducting comparative studies with existing neuroprotective agents and treatments can establish its relative effectiveness and potential advantages; future studies should explore its effects on a broader spectrum of neurodegenerative diseases, such as Alzheimer's, Parkinson's, and multiple sclerosis, to determine its applicability across different conditions; and exploring its potential in combination with other therapeutic agents could lead to synergistic effects and improved outcomes for patients with neurodegenerative diseases.

## Figures and Tables

**Table 1:** Summary of *Moringa oleifera* neuroprotective properties.

Property	Description	Mechanism	References
Neuroprotective agents	Agents that impede the development of injury and neurofunction impairment by preventing changes to the structure and function of the nervous system	Protection against oxidative stress and inflammation, enhancement of neuronal signaling pathways	[63, 64]
Antioxidant properties	Associated with phenolic compounds such as flavonoids, phenols, tannins, and alkaloids; *M. oleifera* exhibits noteworthy antioxidant qualities	Inhibition of lipid peroxidation, enhancement of cellular antioxidant defenses	[44, 45, 49]
Anti-inflammatory effects	*M. oleifera* shows antineuroinflammatory effects attributed to compounds like moringin, astragalin, and isoquercitrin	Suppression of proinflammatory cytokines and increases anti-inflammatory molecules	[54, 55]
Modulation of neurotransmitters	Significant effects of *M. oleifera* on neurotransmitter levels; influences serotonin, dopamine, and acetylcholine	Regulation of neurotransmitter synthesis, release, and reuptake mechanisms	[5, 61]

## Data Availability

The data used to support the study are included within the article.
